# Effectiveness of Multicomponent Balance Training and Sensorimotor Foot Mobilization on Balance and Functional Mobility in Patients With Diabetic Peripheral Neuropathy

**DOI:** 10.7759/cureus.109248

**Published:** 2026-05-19

**Authors:** Rutuja D Bhawad, Suraj Kanase

**Affiliations:** 1 Department of Neurosciences, Krishna College of Physiotherapy, Krishna Vishwa Vidyapeeth (Deemed to be University), Karad, IND

**Keywords:** balance impairment, gait stability, peripheral nerve damage, postural control, proprioception

## Abstract

Background

Diabetic peripheral neuropathy (DPN) is characterized by sensory impairment, reduced ankle mobility, and muscle weakness, leading to balance deficits, gait instability, and an increased risk of falls. Conventional exercise programs used in individuals with DPN primarily focus on general functional improvement and may not sufficiently address the combined sensory and motor deficits responsible for balance impairment. Despite the growing use of multicomponent balance training, evidence regarding its effectiveness in improving balance and functional mobility in individuals with DPN remains limited, highlighting the need for the present study.

Methods

This pre-post experimental study included 64 participants aged 45-65 years diagnosed with DPN. Participants were equally allocated into two groups. Group A received Multicomponent Balance Training, while Group B received Multicomponent Balance Training combined with Sensorimotor Foot Mobilization. The intervention was administered for six weeks, three sessions per week. Outcome measures included the Berg Balance Scale (BBS) to assess balance and the Timed Up and Go Test (TUG) to evaluate functional mobility.

Results

Post-intervention analysis revealed significant improvements in balance and functional mobility in both groups. However, Group B demonstrated significantly greater improvements compared to Group A, with a mean difference of 10.4 points on the BBS (*P* < 0.0001) and 8.25 seconds on the TUG test (*P* < 0.0001), indicating superior enhancement in postural control and mobility.

Conclusions

The addition of sensorimotor foot mobilization to multicomponent balance training is more effective than balance training alone in improving balance and functional mobility in patients with DPN.

## Introduction

According to the World Health Organization (2021), diabetes is one of the fastest-growing global health issues, affecting approximately 536.6 million adults worldwide, with nearly 1 in 10 adults affected and a significant proportion remaining undiagnosed. The prevalence is higher among middle-aged and older adults, particularly after 40 years of age, and it affects both males and females, although slight gender variations have been reported across populations [1]. The most frequently occurring complication associated with diabetes is diabetic peripheral neuropathy (DPN), which is classified as a *length-dependent neuropathy*, typically beginning in the feet and progressing proximally up the legs, with the potential to eventually affect the arms and hands [2]. It is usually associated with the loss of sensory function in the distal ends of the limbs along with the presence of painful sensations, which can create significant disability for the individual with type 1 and type 2 diabetes [1]. As a result of neuropathy, both types of diabetes lead to impaired sensory and motor nerve function, causing loss of protective sensation, reduced proprioception, muscle weakness, and impaired balance control. These deficits increase postural instability and gait disturbances, thereby elevating the risk of falls. Diabetic neuropathy is also associated with foot ulcerations and lower-limb amputations, further contributing to functional limitations and complications in individuals with diabetes [3]. Although less frequently reported in individuals with prediabetes, peripheral neuropathy has been observed in people with type 2 diabetes. Its severity tends to worsen as the disease progresses. There is significant impairment of proprioception, kinesthetic awareness, and muscle strength in the lower limb for an individual with peripheral neuropathy, which increases the risk of being unable to retain one's balance or to perform normal activities of daily living.

DPN, also called distal symmetric polyneuropathy (DSPN), is a type of peripheral nerve disorder that progresses over time and causes impairments in sensory functions, motor control, autonomic nervous system function, and tendon reflexes (i.e., muscular reactions based on a stimulus) [4]. DPN is characterized by progressive distal sensory and motor dysfunction that adversely affects postural stability, gait efficiency, and lower-limb muscle performance [5,6].

Individuals with DPN exhibit reduced proprioception due to damage to peripheral sensory nerves, which impairs the transmission of position and movement sensations from the feet and ankles to the central nervous system [7]. Motor nerve involvement further contributes to muscle weakness and atrophy, particularly in the distal lower limbs, leading to reduced muscle strength [8,9]. Additionally, chronic hyperglycemia and altered biomechanics may cause stiffness of periarticular tissues and limited joint flexibility, resulting in decreased ankle mobility and range of motion. These impairments adversely affect gait, balance, and functional mobility in individuals with DPN. In healthy individuals, postural stability is maintained through integration of sensory, motor, and vestibular inputs [10]. However, impaired somatosensory feedback from the lower extremities in DPN leads to increased postural sway, delayed responses, and poor coordination [11]. These deficits predispose individuals to gait instability and frequent falls, particularly during challenging conditions such as uneven surfaces and low-light environments [11,12]. Studies have demonstrated that balance impairments are significantly greater in individuals with DPN compared to those without neuropathy, with additional gait alterations including reduced gait speed, shorter step length, increased double support time, and greater gait variability [12]. Impaired ankle muscle strength and delayed reflex responses further increase fall risk [13], negatively impacting quality of life and independence.

The somatosensory system plays a vital role in maintaining postural control and body orientation during movement [6]. Somatosensory stimulation enhances the sensitivity of lower-limb sensory receptors, thereby improving sensory feedback, proprioceptive input, and postural control through stimulation of cutaneous and deep receptors in the joints and muscles [14]. Medical treatment for diabetic neuropathy primarily focuses on glycemic control with respect to medication and helps to provide symptomatic relief from pain [15]. However, more than 40% of diabetics who demonstrate good glycemic control will develop some degree of neuropathy. Noninsulin medications are commonly prescribed [16]. Evidence indicates that structured physiotherapy treatment has a large impact on balance and mobility improvement in people with DPN [17].

Conventional physiotherapy for DPN includes range-of-motion exercises, strengthening exercises, wobble board training, functional balance exercises, vestibular exercises, proprioceptive training, and gait training. Systematic reviews and randomized controlled studies have reported that aerobic exercise, flexibility training, and gait training can improve static and dynamic balance, gait performance, and overall postural stability in individuals with DPN [17-19]. Electrotherapeutic modalities such as transcutaneous electrical nerve stimulation (TENS), infrared therapy, and low-intensity laser therapy have also been utilized, with TENS shown to be effective in reducing pain [14]. Sensorimotor training is a therapeutic approach that combines sensory stimulation and motor exercises to improve proprioception, balance, and neuromuscular control. In individuals with DPN, it helps address sensory deficits and impaired ankle control, thereby improving gait and postural stability. Studies have shown that sensorimotor training improves gait, ankle proprioception, and quality of life [19], while foot and ankle mobilization combined with stretching increases joint range of motion in individuals with DPN [20].

The multicomponent balance training model targets multiple physiological systems, including motor control, motor learning, and fall risk factors [21]. It emphasizes functional task practice to enhance balance regulation, neuromuscular coordination, sensory integration, musculoskeletal performance, and motor control [22]. A combination of balance training, endurance training, and resistance training has shown improvements in balance, walking speed, and endurance, along with reducing the displacement of the center of pressure [23]. 

The Michigan Neuropathy Screening Instrument (MNSI), developed by Feldman et al. [24,25], is a standardized and widely used tool for assessing DPN. The MNSI is provided by the Michigan Diabetes Research Center, University of Michigan, and is openly available for research use as per the published guidelines. It was used in accordance with these guidelines. The Berg Balance Scale (BBS) and Timed Up and Go (TUG) test were used as outcome measures, as they are reliable and commonly used clinical tools for assessing balance, functional mobility, and fall risk in individuals with DPN [26,27].

However, limited evidence exists regarding the combined effect of multicomponent balance training and sensorimotor foot mobilization in individuals with DPN, highlighting the need for the present study to evaluate its effectiveness in improving balance performance and reducing fall risk.

## Materials and methods

This pre-post experimental study was conducted over a period of six months in 2025 at Krishna College of Physiotherapy, Karad, Maharashtra, India, after obtaining institutional ethical clearance from Krishna Vishwa Vidyapeeth, Deemed to be University (Ref. No.: KVV/IEC/10/2025). The study was initiated. Written informed consent was obtained from participants with chronic DPN before their voluntary enrollment in the study. Sixty-four participants with chronic DPN were recruited using a convenience sampling method, and the sample size was determined based on sample size calculation. Participants were selected based on predefined inclusion and exclusion criteria, including males and females aged 45-65 years diagnosed with DPN. Participants demonstrating sensory loss, assessed using the MNSI, along with motor impairment and impaired balance assessed through functional balance assessments, were included in the study. Participants who were pregnant, had an open wound around the foot, limb amputation, or cardiac dysrhythmia were excluded from study participation.

Potential confounding variables such as age, gender, duration of diabetes, glycemic control (HbA1c and fasting blood glucose levels), comorbidities, medication use, and baseline functional measures were considered. To reduce potential imbalance, participants were allocated equally into two groups using a non-random allocation method based on convenience sampling, and baseline demographic and clinical characteristics were recorded from patient history and clinical records and compared between groups before intervention.

Group A participants received a program of multicomponent balance training, while those in Group B received multicomponent balance training along with sensorimotor foot mobilization. The intervention protocol was based on previously reported exercise frequencies and durations shown to be effective in improving balance and functional mobility in individuals with DPN [21-23]. The intervention was administered individually, three times per week for six weeks, with each session lasting approximately 40-45 minutes. The protocol included static balance exercises such as tandem standing, single-leg stance, and Romberg stance on stable and unstable surfaces; dynamic balance exercises including weight shifting, reaching tasks, and perturbation training; gait training such as tandem walking, heel-to-toe walking, and obstacle negotiation; and functional task training including sit-to-stand, step-ups, and turning activities. Each exercise was performed for 2 sets of 10 repetitions or maintained for 20 seconds, with rest intervals of 60 seconds between sets. Progression was achieved by reducing the base of support and increasing task complexity. Sensorimotor foot mobilization included passive and active mobilization of the foot and ankle joints using Maitland mobilization techniques for the talocrural and subtalar joints, including dorsiflexion, plantarflexion, and inversion-eversion movements, intrinsic foot muscle activation exercises such as toe curls and short foot exercises, proprioceptive training using textured surfaces and weight-bearing activities, and sensory re-education techniques were applied over the plantar surface of the forefoot, midfoot, heel, and toes using tactile stimulation, textured surfaces, manual graded pressure application, and vibration stimuli. The intervention was administered for 10-15 minutes per session, with repeated stimuli applied for 30 seconds over each region to enhance plantar sensory input and neuromuscular control.

Outcome measures included the BBS and TUG test, which are widely used, validated, and open-access clinical tools for assessing balance and functional mobility [26,27]. All assessment tools were used in accordance with their respective guidelines for research purposes. Assessments were taken before the beginning of the six-week program and at its completion.

The data were entered into an Excel spreadsheet, tabulated, and analyzed using GraphPad InStat (version 3.06; GraphPad Software Inc., San Diego, CA). Descriptive statistics, including mean and standard deviation (SD), were calculated for continuous variables. Normality of data was assessed using the Kolmogorov-Smirnov test. Paired t-tests were used to analyze within-group differences between pre- and post-intervention values, while independent (unpaired) t-tests were used to compare between-group differences. The chi-square test was used for categorical variables. A 95% confidence interval was considered, and a *P*-value of <0.05 was considered statistically significant.

## Results

Baseline demographic and clinical characteristics of the participants are presented in Table 1. A total of 64 participants were included, with 32 (50%) participants in each group: Group A received multicomponent balance training, and Group B received multicomponent balance training combined with sensorimotor foot mobilization.

**Table 1:** Baseline demographic and clinical characteristics of the participants. Values expressed as mean ± standard deviation or frequency (%). Other comorbidities include conditions such as dyslipidemia, thyroid disorders, and cardiovascular diseases. Medication - oral refers to oral antidiabetic drugs. An independent t-test was used for continuous variables, and a chi-square test was used for categorical variables. BBS, Berg Balance Scale; TUG, Timed Up and Go

Variable	Group A	Group B	Test statistics	*P*-value
Age (years)	56.2 ± 5.98	56.2 ± 5.98	*t* = 0.01	>0.05
Gender				
Male	18 (56.3%)	12 (37.5%)	*χ*² = 2.26	>0.05
Female	14 (43.8%)	20 (62.5%)		>0.05
Duration of diabetes (years)	8.4 ± 3.2	8.9 ± 3.6	*t* = -0.59	>0.05
HbA1c (%)	7.8 ± 1.1	8.0 ± 1.2	*t* = -0.69	>0.05
Fasting blood sugar (mg/dL)	156.3 ± 28.5	162.7 ± 30.1	*t* = -0.87	>0.05
Hypertension				
Yes	14	16	*χ*² = 0.25	>0.05
No	18	16		>0.05
Other comorbidities				
Yes	10	12	*χ*² = 0.28	>0.05
No	22	20		>0.05
Medication				
Oral	20	18	*χ*² = 0.39	>0.05
Insulin	6	8		>0.05
Both	6	6		>0.05
BBS (Pre)	26.8 ± 4.06	27.6 ± 3.76	*t* = -0.82	>0.05
TUG (Pre)	27.2 ± 2.01	28.5 ± 1.60	*t* = -1.83	>0.05

In Group A, 18 (56.3%) participants were male, and 14 (43.8%) were female, while in Group B, 12 (37.5%) were male and 20 (62.5%) were female. The mean age of participants was 56.2 ± 5.98 years in both groups (estimated from the grouped age distribution).

Clinical characteristics, including duration of diabetes, glycemic control (HbA1c and fasting blood glucose levels), comorbidities, and medication use, were also recorded and compared between groups. Baseline functional measures showed that the mean BBS score was 26.8 ± 4.06 in Group A and 27.6 ± 3.76 in Group B, while the mean TUG time was 27.2 ± 2.01 seconds in Group A and 28.5 ± 1.60 seconds in Group B.

The minimal clinically important difference (MCID) was considered while interpreting the outcomes, with an MCID of 4-7 points for the BBS and approximately 3 seconds for the TUG test [28].

No statistically significant differences were observed between the groups for the measured baseline variables (*P* > 0.05). Nevertheless, as not all potential confounding factors were evaluated, residual confounding cannot be ruled out.

**Figure 1:**
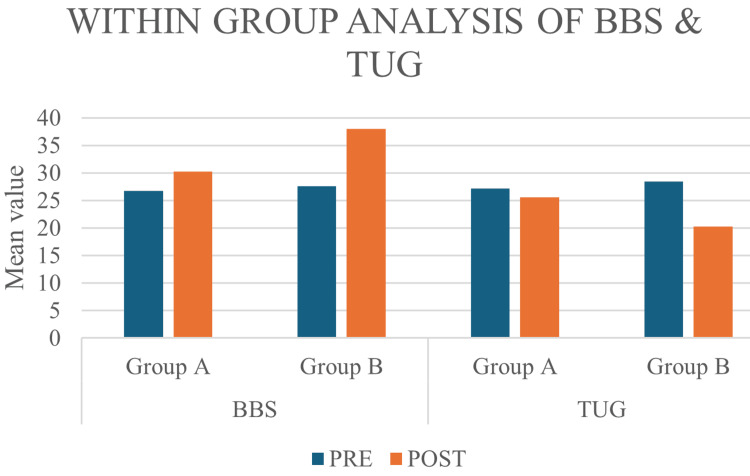
Comparison of pre- and post-intervention scores within the groups. BBS, Berg Balance Scale; TUG, Timed Up and Go

Within-group analysis demonstrated significant improvements in balance and functional mobility following the six-week intervention in both groups. In Group A, BBS scores showed a statistically significant improvement from pre- to post-intervention (*P* = 0.001), indicating improvement in balance (Table 2). Group B demonstrated a significant improvement in BBS scores following intervention (*P* < 0.0001), with a greater mean change compared to Group A (Table 2). For functional mobility, within-group analysis of the TUG test showed statistically significant improvement in Group A (*P* = 0.0047) and Group B (*P* < 0.0001) following the intervention (Table 2, Figure 1). The reduction in TUG time was greater in Group B than in Group A, with Group B demonstrating a mean improvement of 8.25 seconds compared to 1.53 seconds in Group A, indicating superior improvement in functional mobility following the intervention.

**Table 2:** Comparison of pre- and post-intervention scores within groups (Group A and Group B). BBS, Berg Balance Scale; TUG, Timed Up and Go; SD, standard deviation

Outcome measure	Group	Phase	Mean	SD	*t*-value	*P*-value	Result	Mean difference
BBS	Group A	Pre	26.8	4.06	3.41	0.001	Very significant	3.43
		Post	30.2	3.99				
	Group B	Pre	27.6	3.76	9.54	<0.0001	Extremely significant	10.4
		Post	38	4.88				
TUG	Group A	Pre	27.2	2.01	2.93	0.0047	Very significant	-1.53
		Post	25.6	2.16				
	Group B	Pre	28.5	1.60	20.62	<0.0001	Extremely significant	-8.25
		Post	20.3	1.59				

The observed improvements in BBS and TUG scores exceeded the reported MCID values of 5 points and 8 seconds, respectively, indicating clinically meaningful improvement following the intervention [28].

The graphical representation of within-group BBS and TUG changes is shown in Figure 1. 

**Figure 2:**
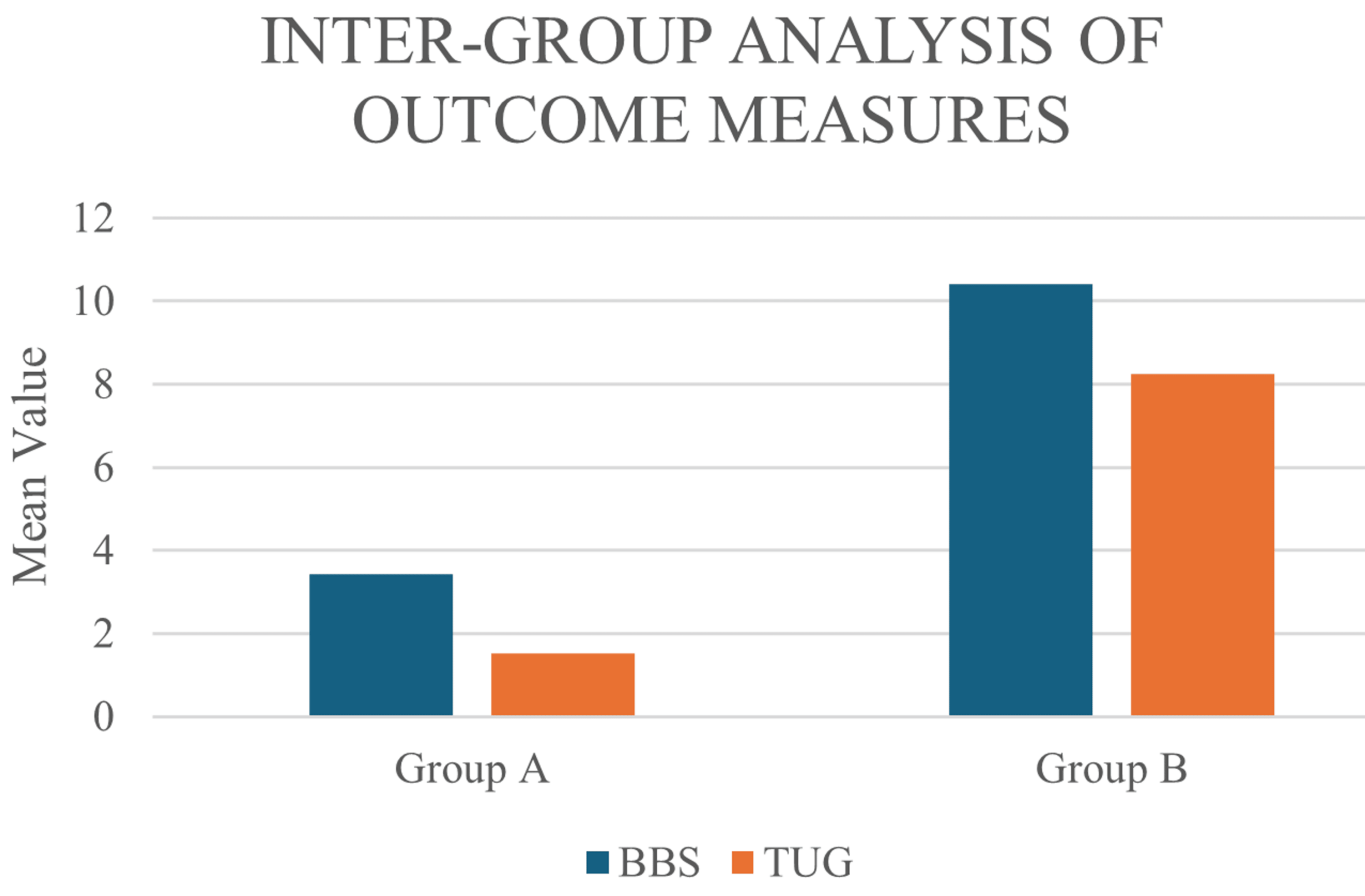
Comparison of intervention scores between the groups. BBS, Berg Balance Scale; TUG, Timed Up and Go

Between-group comparison of BBS and TUG scores revealed a statistically significant difference favoring Group B over Group A following the intervention period (*P* < 0.0001), indicating improved balance and Functional mobility performance in participants who received sensorimotor foot mobilization in addition to balance training (Table 3, Figure 2). The graphical representation of intergroup changes in BBS and TUG scores is shown in Figure 2.

**Table 3:** Inter-group analysis of BBS and TUG between Group A and Group B. Mean difference represents the difference between Group A and Group B post-intervention changes. BBS, Berg Balance Scale; TUG, Timed Up and Go; SD, standard deviation; *N*, number of participants.

Outcome measure	Group	N	Mean	SD	Mean difference	*t*-value	*P*-value	Result
BBS	Group A	32	3.43	1.29	6.969	20.96	<0.0001	Extremely significant
	Group B	32	10.4	1.36				
TUG	Group A	32	1.53	0.56	6.71	52.95	<0.0001	Extremely significant
	Group B	32	8.25	0.43				

## Discussion

This study demonstrated that multicomponent balance training combined with sensorimotor foot mobilization improved balance and functional mobility in individuals with DPN. The training program for this study included each participant having the opportunity to participate in individualized training and therapy sessions with the intention of improving their balance, strengthening their feet, improving their stability, stimulating the body to develop proprioception, and improving the subject's overall motor control. The training and rehabilitation process involved each subject's services to be provided with the use of balance training, foot strengthening, stability, and sensorimotor rehabilitation exercises to determine the effectiveness of the training protocol. Participants who received the combined intervention showed greater improvement in BBS and TUG scores compared to those who received multicomponent balance training alone, indicating the additional benefit of incorporating sensorimotor foot mobilization into rehabilitation programs for DPN.

The improvement in BBS scores observed in the present study suggests enhancement in both static and dynamic balance, consistent with previous evidence demonstrating that structured balance training stimulates proprioceptive feedback, enhances neuromuscular coordination, and optimizes postural muscle activation [17]. Similarly, reductions in TUG completion times reflect enhanced dynamic balance and functional mobility, in line with evidence from multicomponent exercise interventions. A meta-analysis reported that multicomponent programs significantly reduce TUG time. The observed improvements in BBS and TUG scores also exceeded the reported MCID values, indicating clinically meaningful improvement following the intervention.

The findings of the present study may be explained by the important role of plantar sensory input and foot musculature in postural control and balance regulation. Cutaneous mechanoreceptors contribute significantly to postural reflexes necessary for balance recovery [29]. The feet function as the primary interface between the body and the supporting surface by providing sensory feedback regarding environmental and surface conditions [30]. Somatosensory input from the plantar skin, muscles, ligaments, and joints contributes to appropriate postural adjustments [31]. Activation of the foot and ankle musculature is also essential for maintaining postural stability against external perturbations.

The results of the present study suggest that improving cutaneous feedback from the plantar surface may be a promising approach for managing balance and sensory impairments in individuals with DPN. The feet play a fundamental sensory role in postural control during standing and movement. Plantar cutaneous mechanoreceptors transmit tactile and proprioceptive input to the central nervous system, enabling continuous postural adjustments and improved stability [32]. This sensory feedback also facilitates activation and coordination of the intrinsic foot muscles, which help maintain the medial longitudinal arch, support dynamic foot function, and regulate foot support during movement [32,33]. Together, these sensory and muscular mechanisms may contribute to improved balance and functional mobility in individuals with DPN. Ahmad et al. similarly reported significant improvements in TUG performance, proprioception, and postural stability following an eight-week sensorimotor training program in older adults with DPN [34]. The findings of the present study further reinforce existing evidence by demonstrating that combining sensorimotor foot mobilization with multicomponent balance training may provide greater improvements in balance and functional mobility than balance training alone.

Several studies have demonstrated that tailored sensorimotor and comprehensive balance interventions can improve postural control, functional mobility, and quality of life in individuals with DPN. A single-blind randomized controlled trial (RCT) involving 40 patients reported that a six-week sensorimotor training program combined with education resulted in improvements in spatiotemporal gait parameters, ankle joint proprioception, and quality of life compared to education alone [19]. These findings suggest that sensorimotor interventions may enhance sensory feedback and gait performance in individuals with DPN. Similarly, another RCT involving middle-aged and older adults with DPN demonstrated that an eight-week sensorimotor training program improved static and dynamic balance, proprioceptive accuracy, TUG performance, and center of pressure (COP) sway measures, with significant effects observed across most outcomes [34,35].

The results of this study suggested that combining sensory feedback from the foot with precise manual mobilization of foot mechanoreceptors and targeted strength training may significantly enhance patients’ balance and functional mobility. Additionally, a multicenter RCT using Feldenkrais-based sensorimotor training in older adults with DPN showed significant short-term improvements in postural control, dynamic balance, and fear of falling, with sustained benefits in balance and quality of life at three months. Similarly, a large RCT in 150 elderly patients with DPN found that a 12-week multifactorial balance program significantly improved postural stability, reduced fall risk, and enhanced quality of life [36].

Overall, these findings support the effectiveness of both sensorimotor training and multicomponent balance programs in improving balance, gait, and reducing fall risk in individuals with DPN.

The synergistic effect observed in this study may be attributed to the integration of balance training with sensorimotor foot mobilization. Balance training primarily strengthens neuromuscular control and functional strength, whereas foot mobilization enhances plantar sensory input, joint mobility, and ankle mechanics, leading to better proprioceptive feedback and weight distribution during functional tasks. This integrated approach addresses both sensory and motor deficits characteristic of DPN, resulting in greater functional gains compared to interventions focusing on a single modality.

Balance confidence was one of the functional measures that was enhanced through the intervention, which is a critical factor affecting daily functional activity performance. Low balance confidence has been found to relate to an increased postural instability level, decreased level of mobility, and an increased probability of falling in people with DPN [37]. Improvements in postural control and functional mobility demonstrated in this study may have helped to improve patients' confidence while engaging in daily functional activities requiring balance, thus facilitating their ability to engage fully in their daily activities without fear of falling.

The combination of multicomponent balance training and sensorimotor foot mobilization positively impacted both physical performance and the confidence of participants in completing balance tasks. This implies that putting sensory and motor components together can provide significant benefit to individuals with DPN in their pursuit of safe mobility and effective independence.

According to research, those suffering from DPN improve their balance and mobility after six weeks of balance training and sensorimotor foot exercise. The standard exercise group displayed moderate improvement; however, the combined group saw significantly improved balance and mobility, both clinically and statistically. This indicates that both types of exercise are beneficial, but the combination of both types of exercise is a more effective means of improving balance and mobility.

The success of the experimental group could be due to the combination of different methods used to treat their sensory and motor deficits. By using multiple methods of treating both types of deficits at the same time, they had the best chance for improving their postural control and functional performance; therefore, multicomponent balance training and sensorimotor foot mobilization would be good clinical treatment options for individuals who have DPN to improve balance and functional mobility.

There are specific limitations acknowledged in this study. The intervention duration was limited to six weeks, and long-term follow-up was not performed; therefore, the long-term sustainability of treatment effects could not be determined. Additionally, the use of convenience sampling may limit the generalizability of the findings. Future studies should include randomized study designs and longer follow-up periods to evaluate long-term functional outcomes.

The addition of sensorimotor foot mobilization to multicomponent balance training demonstrated greater improvements, suggesting a beneficial combined effect on neuromuscular control and functional performance. This approach is cost-effective, clinically applicable, and can be easily integrated into routine physiotherapy practice, offering a practical strategy for rehabilitation in this population.

## Conclusions

In this study, we concluded that multicomponent balance training combined with sensorimotor foot mobilization significantly improved balance and functional mobility in individuals with DPN. Participants who received the combined intervention demonstrated greater improvement in BBS and TUG scores compared to those who received multicomponent balance training alone.

The findings suggest that while conventional exercises can enhance functional outcomes, combining balance training with sensorimotor foot mobilization more effectively addresses both sensory and motor deficits, leading to better postural control, proprioception, and overall functional performance. This study provides evidence to support the clinical implementation of such integrated rehabilitation programs in patients with DPN, with the potential to reduce fall risk and improve mobility.
